# Atropine Eye Drops in Childhood Myopia Control: A Review

**DOI:** 10.7150/ijms.126651

**Published:** 2026-03-09

**Authors:** Qin Zhu, Yanhua Chen, Xuejiao Li, Liping Xue, Xiaofan Zhang, Guanglong Zhou, Yuan Zhou, Jieying Zhang, Allison Wilde, Yingting Zhu, Yuezu Li

**Affiliations:** 1 Department of Pediatric Ophthalmology, Affiliated Hospital of Yunnan University, Kunming 650021, China.; 2 Department of Ophthalmology, Affiliated Hospital of Yunnan University, Kunming 650021, China.; 3 Department of Refractive Surgery Center, Affiliated Hospital of Yunnan University, Kunming 650021, China.; 4 BioTissue (Tissue Tech Inc), Miami, Florida 33126, USA.

**Keywords:** atropine, myopia, axial length, spherical equivalent, rebound

## Abstract

Myopia is a pandemic problem in this world. Per 2019 report of the World Health Organization in their first-ever report on vision, ~27% world's population are suffering from myopia. This trend is expected to increase to ~50% by 2050. Unfortunately, no cure method is available so far due to its complications of the mechanistic actions. Therefore, control of myopia is the focus clinically. Currently, it is suggested that 0.05% atropine is optimal, balanced in efficacy and rebound in controlling myopia initiation and progression. In such a control process, measurement of reduction in spherical equivalent refraction (SER) progression and axial length (AL) elongation are critical for efficacy and rebound of atropine eye drops. For example, in Low-concentration Atropine for Myopia Progression (LAMP) study over one year, 0.05%, 0.025%, and 0.01% atropine can reduce SER progression by 67%, 43%, and 27%, decrease AL elongation by 51%, 29%, and 12%, respectively, while 0.5%, 0.1%, and 0.01% atropine may increase rebound by 68.4%, 58.9%, and 24.3%, respectively. Mechanistically, atropine, a muscarinic receptor antagonist, is used as the primary medication for controlling myopia due to its effectiveness and affordability.

In this review, we provide a concurrent view of myopia epidemiology, atropine use to control myopia, its tentative mechanism, balance of its effects as well as side effects, and its clinical application methods in hope for clinical ophthalmologists to effectively control this problematic disease worldwide.

## 1. Introduction: The Global Myopia Epidemic

Myopia (nearsightedness) is characterized by increased axial length (AL) of the eye, and this causes the refractive error known as “nearsightedness” 1 due to increased anterior-posterior diameter of the eye relative to refracting power of cornea and lens, distorted image focal point and blurred image in front of retina 2 (also reviewed in 3-8). Technically, myopia is defined as the spherical equivalent of refractive error < -0.50 diopter (D) but > -5.00 D, high myopia as a spherical equivalent of refractive error ≤ -6.00 D in one eye 9.

Childhood myopia has evolved into a global public health crisis of unprecedented scale. Epidemiologically, ~50% people in the world will suffer myopia by 2050, with ~10% of high myopia (≤ -6.00 diopters) 10. In some East Asian regions, nearly 90% of secondary-school children have myopia, with ~20% of high myopia 11, substantially increasing their risk of sight-threatening complications such as retinal detachment, myopic maculopathy, glaucoma, and cataracts 12.

Although there are significant differences of myopia occurrence among different regions, with East Asia having the highest rates (80-90% in teens), myopia is a global epidemic with rising rates in Europe, North America (around 42%), and developing nations, though lower than in Asia. South America, Africa, and parts of South Asia show lower, but still increasing, prevalence, demonstrating a global rise driven by lifestyle changes like increased near work and less outdoor time, making it a major public health concern worldwide 13.

The economic burden is staggering, encompassing direct costs of optical correction, medical management of complications, and immeasurable impacts on quality of life and productivity. In high-prevalence regions of East Asia, myopia onset now commonly occurs between 6-8 years of age, with rapid progression during primary school years 14. A clinical investigation shows 0.01%-0.05% atropine satisfactorily controls myopia progression in East Asia regions 15. This early onset is particularly concerning given the strong association between younger age at onset and eventual progression to high myopia. Consequently, effective interventions to slow down myopia progression in childhood have become one of the fastest-growing areas of ophthalmic research and clinical innovation, with low-dose atropine emerging as a cornerstone of pharmacological management.

Myopia's economic impact is massive, spanning billions in direct correction costs (glasses, contacts, surgery) and huge indirect losses from lost productivity, with projections hitting $229 billion by 2050 due to rising prevalence, especially high myopia linked to serious complications like glaucoma. Controlling progression saves money by reducing lifelong care and preventing costly, vision-threatening conditions, proving management is cheaper than treatment, even with upfront costs for interventions like specialized lenses or atropine, highlighting the societal benefit of prevention 16.

Myopia stems from a mix of genetics (runs in families) and environment, with key risk factors being lots of near work (screens, reading) and not enough outdoor time, which exposes eyes to bright light that slows eye growth. Factors like ethnicity (higher in Asians), early hyperopia, and certain binocular vision issues also increase risk, while spending over 90 mins outdoors daily significantly lowers onset risk, though its effect on progression after onset is less clear 17.

Atropine eyedrops are now being used for prevention of the onset of myopia in pre-myopic children with significant effect. For example, a graduated-concentration atropine protocol (0.025%, 0.05%, 0.125%) achieved satisfactory outcomes, defined as prevention of myopia onset and maintenance of axial elongation below the predefined progression threshold, in 64% of premyopic children at 2-year follow-up 18, a concentration of 0.025% atropine resulted that 21% of children in atropine group suffered myopia onset compared to 54% in control group in one year, indicating effectiveness in preventing myopia onset 19. A case-control study demonstrated that 0.01% atropine group progressed -0.31D / 0.12mm compared to -0.76D / 0.21mm in the control group in one year and the atropine group progressed -0.60D / 0.21mm compared to -1.75D / 0.48mm in the control group in two years 20. A crossover trial in China demonstrated that in 0.01% atropine group, myopia progressed -0.15D and 0.17mm compared to -0.34D and 0.28mm in placebo group in a 1-year study 21. In LAMP2 Clinical Trial, nightly use of 0.05% atropine eye drops significantly reduced the 2-year cumulative incidence of myopia to 28.4%, compared to 53.0% respectively in the placebo group. This study demonstrated that 0.05% atropine is effective in preventing early myopia onset 22.

## 2. Pharmacological Profile and Putative Mechanisms of Action

### 2.1 Basic Pharmacology

Atropine, a non-selective muscarinic antagonist derived from the deadly nightshade plant *Atropa belladonna*, has a complex pharmacological profile that extends beyond its classical effects on pupil dilation (mydriasis) and accommodation paralysis (cycloplegia). When administered in low concentrations (typically 0.01% to 0.05%), it exerts biological effects at concentrations several orders of magnitude below those traditionally used for cycloplegic refraction or uveitis treatment. The molecular basis of its action involves competitive inhibition of acetylcholine at muscarinic receptors (M1-M5), though its affinity varies across receptor subtypes and tissue distributions 23.

Muscarinic acetylcholine receptors (M1-M5) are widely distributed throughout the central nervous system (CNS) and peripheral nervous system (PNS), particularly in organs innervated by parasympathetic neurons. Their specific distribution dictates their physiological function 24. Importantly, atropine's ocular pharmacokinetics differ significantly according to its concentration. Low-concentration formulations may achieve minimal systemic absorption due to the minute quantities delivered (approximately 5-50 µg per drop), resulting in negligible serum concentrations and thus an excellent systemic safety profile. However, local ocular effects, particularly on retinal and scleral signaling, remain profound even at these ultra-low doses.

### 2.2 Evolving Understanding of Mechanisms

The precise mechanisms through which atropine slows myopia progression have been extensively investigated yet remain incompletely characterized (reviewed in 7, 25). Earlier hypotheses centered on accommodation blockade, positing that by paralyzing the ciliary muscle, atropine might eliminate accommodative lag and its associated hyperopic defocus on the peripheral retina - a putative stimulus for axial elongation. However, animal studies have fundamentally challenged this view.

#### Non-accommodative Pathways

Intravitreal injection of atropine in animal models effectively inhibits axial elongation without affecting accommodative amplitude or pupillary constriction, demonstrating that its anti-myopic effects occur independently of accommodation 26. The non-accommodative pathways include:

**Muscarinic Receptors:** Gene expression studies in myopic animal models reveal that atropine administration significantly upregulates muscarinic M1, M3, and M4 receptors in the sclera while leaving M2 and M5 largely unchanged. This receptor-specific pattern suggests targeted effects on scleral remodeling pathways 27.

The muscarinic receptors belong to G-protein coupled receptors (GPCRs), which might be activated by neurotransmitter acetylcholine 28, 29. GPCRs activation proceeds with ligand binding, activating phospholipase C, inducing second messengers or diacylglycerol (DAG) and inositol triphosphate (IP3) 28, 29. DAG and IP3 might then promote protein kinase C activation and calcium release intracellularly to induce signaling cascade 28, 29. Alternatively, induction of inhibitory Gi-coupled receptors might lead to reduction of adenylyl cyclase, and thus protein kinase A, leading to cellular cyclic adenosine monophosphate inhibition and thus inhibitory cellular responses 28, 29. Among muscarinic receptors, M1, M3, and M5 are stimulatory while M2 and M4 are inhibitory 29, 30.

**Scleral Remodeling:** Atropine appears to modulate the extracellular matrix composition of the sclera. In progressive myopia, scleral fibroblasts demonstrate reduced proliferation and downregulation of type I collagen and glycosaminoglycans (GAGs), leading to biomechanical weakening and ocular elongation. Mechanistically, atropine reduces myopia progression through increasing collagen synthesis and thus scleral thickness in the stromal layer 31.

During axial length elongation, structural changes occurred in myopia onset and progression 32. In this process, sclera thinning arose primarily at the posterior pole 33. Scleral remodeling relies on the changes of the scleral extracellular matrix (ECM), which plays a critical role in thinning of the sclera 34, 35. During myopia progression, scleral collagen is steadily decreasing, leading to its progressive breakdown 36. In addition, sclera proteoglycan is also steadily decreasing 37. As a result, scleral fibril assembly is disrupted, and the tissue biomechanics deteriorate 38. In summary, the results indicate that scleral structural changes contributes to myopia progression by scleral ECM remodeling (reviewed in 39).

Scleral remodeling in myopia involves the following molecular pathways that mediate the synthesis as well as degradation of scleral extracellular matrix (ECM), particularly collagen. Key factors and signaling pathways and factors include the following: 1. Hypoxia-Inducible Factor-1α (HIF-1α) Signaling: Scleral tissue might become hypoxic (oxygen-deficient) in case of myopia because of decreased choroidal blood flow, which induces HIF-1α to promote trans-differentiation from fibroblasts to myofibroblasts and decreases the synthesis of type I collagen. This process leads to scleral thinning and weakening. 2. Matrix Metalloproteinases (MMPs) and Tissue Inhibitors of Metalloproteinases (TIMPs) Involved: Scleral remodeling is mediated by the balance of ECM-degrading enzymes (MMPs, such as MMP-2 and MMP-9) and their inhibitors (TIMPs, such as TIMP-2). In myopic sclera, MMP activity increases while TIMP activity decreases, causing excessive collagen degradation and decreased collagen fiber content. 3. Transforming Growth Factor-β (TGF-β) Signaling: The TGF-β family plays a significant role. For example, decreased levels of TGF-β subtypes, such as TGF-β1, are associated with reduced collagen synthesis, whereas increased levels of other types, such as TGF-β2, may contribute to scleral thinning by promoting degradation. This signaling interacts with endoplasmic reticulum (ER) stress and the Wnt pathways. 4. Wnt/β-catenin Signaling: Activation of Wnt signaling is associated with myopia progression by promoting scleral fibroblasts to myofibroblasts and decrease of COL-1 synthesis. 5. cAMP Pathway: cAMP signaling is linked to mediating eye growth. Increased cAMP is linked to inhibition of eye elongation through decreasing MMP-2 and increasing collagen synthesis. 6. YAP Signaling: Mechanical stiffness from ECM plays a significant role. Decrease of scleral stiffness in myopia is associated with decreased expression and nuclear translocation of YES-associated protein (YAP), which normally enhances COL1A1 expression. 6. Inflammatory and Other Factors: Pathways involving inflammatory cytokines such as IL-6, TNF-α, Sonic Hedgehog (Shh) pathway, Retinoic Acid (RA), as well as microRNAs also influence the complex regulation of scleral remodeling. In summary, these pathways ultimately cause scleral thinning, disorganizing collagen fibrils, and decreasing biomechanical strength in myopic eyes, and facilitating axial elongation (reviewed in 39).

**Retinal and Choroidal Signaling:** Emerging evidence indicates that atropine increases choroidal thickness and choroidal blood perfusion pressure, potentially improving oxygenation of retinal tissues and counteracting hypoxia-driven ocular growth 40. However, this conclusion is controversial because a recent report suggested that low dose atropine did affect choroidal thickness 41. Additionally, atropine influences dopaminergic signaling in the retina, enhances GABAergic inhibition of ocular growth, and modulates inflammatory cytokine profiles in the retina and retinal pigment epithelium 42, probably through binding to mAchR on the cells to promote release of dopamine, thus inhibiting myopia progression 43, 44.

The current consensus suggests that atropine's anti-myopic effects result from a multifactorial orchestration of receptor-mediated events across multiple ocular tissues rather than a single unified mechanism. This complexity may explain why individual treatment responses vary and why combination therapies targeting multiple pathways simultaneously show promise.

The most well-studied pathway in illustrating atropine's mechanism of action is the competitive antagonist of muscarinic acetylcholine receptors. This core mechanism is the central mechanism to its primary clinical applications. Atropine binds to and inhibits muscarinic acetylcholine receptors, competitively blocking the effects of acetylcholine and other choline esters 7, 45, 46. Such receptors include M1, M2, M3, M4, and M5 receptor subtypes 47. Atropine inhibits effects of acetylcholine on postganglionic cholinergic nerves in tissue such as central nervous system, cardiac smooth muscle, and exocrine glands. It also acts in less innervated smooth muscle that responds to endogenous acetylcholine 45, 48. The actions of atropine can be overcome by increasing the concentration of acetylcholine at receptor sites (for instance, the use of anticholinesterase agents that inhibit the hydrolysis of acetylcholine) 7. Other mechanisms include, for example: 1. Atropine can act through cycloplegic action on smooth ciliary muscles to reduce accommodative eye reactions; 2. Atropine might promote choroidal thickening by ocular growth mechanistically (reviewed in 7).

In summary, the major myopia control mechanism is summarized as follows.

## 3. Clinical Efficacy Across Concentrations

### 3.1 Landmark Clinical Trials

The effect of atropine concentrations on myopia progression has been extensively investigated in several randomized controlled assessments, which sheds light on our clinical practice (reviewed in 53):

ATOM Studies (Singapore): The pioneering ATOM1 Trial demonstrated that 1% atropine produced a remarkable 80% reduction in myopia progression compared to placebo over two years. Nevertheless, the dose at 1% concentration results in significant side effects, such as near vision blur and photophobia, closely related to a profound rebound side effect (~ -1.2 D) when discontinued 54. In ATOM 1 Trial, axial length increased by 0.02 ± 0.35 mm in 1% atropine group after two years 55. By contrast, axial length increased by 0.38 ± 0.38 mm in the control group 55. After three years, axial length increased by 0.29 ± 0.37 mm in 1% atropine group in contrast to 0.52 ± 0.45 mm from the control group 56. The subsequent ATOM2 Trial compared lower concentrations (0.5%, 0.1%, and 0.01%), finding that 0.01% atropine offered the optimal balance of efficacy (approximately 50-60% reduction in progression), minimal side effects, and negligible rebound after cessation 57. In subsequent ATOM 2 Trial, the researchers found that the effectiveness of atropine was concentration dependent, based on axial length increase (by 0.27 ± 0.25 mm, by 0.28 ± 0.28 mm and by 0.41 ± 0.32 mm in 0.5%, 0.1% and 0.01% atropine groups respectively 58, 59. High concentration, for example, 1% atropine, was profoundly more effective than low concentration atropine, for instance, 0.01% atropine, to inhibit axial length elongation by as much as 70% and 94% in a few clinical trials 54, 55, 59-63. Currently, the ATOM3 Study, a randomized clinical trial enrolled children aged 5 to 9 years old, with one or two myopic parents and spherical equivalent refraction of +1.00 to -1.50, is still underway using 0.01% atropine eyedrops to prevent the onset or delay the progression of myopia in school children.

Long-term (10-20 years) follow-up data from ATOM participants, especially in Atropine Treatment Long-term Assessment Study (ATLAS), have confirmed the excellent safety profile of low-concentration atropine, with no increased incidence of cataracts, glaucoma, or other visually significant complications such as macular degeneration 64. The drawback is that while no rebound effect after withdrawal of atropine treatment, myopic progression still occurs as adults, without overall changes of final refractive errors 64.

LAMP Study (Hong Kong): This rigorous RCT compared 0.05%, 0.025%, and 0.01% atropine over two years in children aged 4-12 years. Results demonstrated a clear concentration-dependent effect: 0.05% atropine reduced progression by 67% (spherical equivalent) and 51% (axial length), significantly outperforming both lower concentrations 65. Notably, the 0.01% formulation showed only modest efficacy (27% reduction in SE progression), challenging the ATOM2 findings. The LAMP2 Extension further demonstrated that delayed initiation of 0.05% atropine in previously untreated children still produced substantial benefits, whereas switching from 0.01% to placebo resulted in accelerated "catch-up" progression 22. In the subsequent five-year LAMP Phase 4 Study, the authors showed that treatment with 0.05% atropine effectively controlled myopia, and that re-treatment with 0.05% atropine remained effective after a period of treatment discontinuation 66.

In summary, the LAMP Study provides strong evidence that 0.05% atropine is the optimal concentration for myopia control among the tested doses, demonstrating a significant reduction in myopia progression and superior performance compared to lower concentrations like 0.01%.

### 3.2 Comparative Efficacy Analysis

More recent studies have refined our understanding of concentration-response relationships:

0.05% Concentration: The MOSAIC Trial (Europe) confirmed that 0.05% atropine significantly reduced progression compared to placebo over 24 months, even in older children (mean age 13.9 years) 67. After three years, children who switched from placebo to 0.05% atropine experienced less progression and less axial elongation than those who switched from 0.01% atropine to placebo, highlighting its robust efficacy even when initiated late 67. Similarly, a 2024 prospective study of 0.03% atropine demonstrated significant reductions in both spherical equivalent progression and axial elongation 68.

0.01% Concentration: Efficacy findings for this concentration have been somewhat inconsistent across trials. While ATOM2 reported a significant reduction in progression in two years, the LAMP Study found only a less reduction, and the LAMP2 Prevention Trial showed no significant difference from placebo in delaying myopia onset 22, 69, 70. A 2023 U.S. Trial (n=489) provided further clarification: 0.01% atropine produced consistent and statistically significant slowing of both refractive shift and axial elongation over three years, though the magnitude of effect was more modest than higher concentrations 71. This concentration appears most suitable for younger children with mild progression or as maintenance therapy after initial control with stronger concentrations.

The efficacy of different atropine concentrations may vary across populations and study regions. In particular, the difference in concentration-dependent efficacy between Eastern and Western populations are especially evident in the following: **Higher Efficacy in Asian Populations:** Many studies, including ATOM and LAMP Trials in Hong Kong and Singapore, have indicated greater efficacy of atropine with various concentrations for East Asian children, compared to white or South Asian populations. For example, meta-analyses consistently demonstrate a greater pooled treatment effect of atropine in East Asian populations compared with White populations 72. **Optimal Concentration Difference:** For East Asian children, concentrations of 0.05% atropine are indicated as optimal. Higher concentrations (for example, 0.5% or 1%) of atropine show maximal efficacy, which are associated with more pronounced adverse effects and rapid myopia rebound after atropine cessation 73. For white populations, the efficacy of 0.01% atropine was modest or not significant in several studies (for example, the PEDIG study in the US), while some other studies in Spain and USA have concluded its effectiveness with minimal side effects. More pronounced side effects, such as photophobia and reading difficulty, have been reported in concentrations like 0.05% in Caucasian populations compared with Asian children 68, 74. **Factors Affecting Mechanisms:** Baseline Myopia Progression: Asian populations exhibit faster baseline myopia progression as well as greater axial elongation than European populations, which might cause greater treatment effect 75. Genetic Factors: Genetic predispositions might affect individuals respond for atropine treatment 76. **Environmental Factors:** Environmental factors, such as elevated educational pressure and less outdoors activities in some Asian countries, also affect the progression rates and treatment response 77. **Physiological Differences:** East Asian populations have greater photopic pupil dilation on atropine treatment, compared to white Europeans related to iris pigmentation differences 77. **Overall Efficacy**: Atropine retards myopia progression more effectively in Asian populations, especially in East Asian populations than in South Asian populations or white people 77. **Optimal Concentration:** For East Asian children, 0.01% atropine is considered as effective while 0.05% atropine considered as optimal concentration 70, while in white European children, 0.01% atropine often does not show much effectiveness. The optimal concentration for Western children remains unclear 78, 79. **Side Effects:** Side effects, such photophobia and reading difficulties, are more pronounced in Caucasian populations than Asian children 68. **Pupil Dilation:** East Asian populations exhibit greater photopic pupil dilation by atropine compared to white European populations. This difference might be due to differences of iris pigmentation and genetic factors 77.

Notes: Eligibility Criteria for Above Trials/Investigation:

**1.** Eligibility criteria for LAMP (1 Year), Hong Kong 65 (atropine 0.01%, 0.025%) were children aged 4-12 years with myopic refraction > 1.0 D in both eyes, astigmatism of < 2.5 D, and documented myopic progression of > 0.5 D in the past one year were enrolled. Excluded were those also with ocular diseases (e.g., cataract, congenital retinal diseases, amblyopia, and strabismus), previous use of atropine, pirenzepine, orthokeratology lens, or other optical methods for myopia control, allergy to atropine, or systemic diseases (e.g., endocrine, cardiac, and respiratory diseases). **2.** Eligibility criteria for U.S. Trial (3 Years) 71 were enrolled participants with -0.50 diopter (D) to -6.00 D spherical equivalent refractive error (SER) and no worse than -1.50 D astigmatism. **3.** Eligibility criteria for Lithuania 68 were Caucasian children aged 6-12 years with a cycloplegic SE (sphere plus half of the cylinder power) refraction ranging between -1.0 D and -5.0 D and astigmatism and anisometropia <1.5 D. Exclusion criteria included prior use of atropine eye drops or other myopia progression control, congenital or chronic ocular diseases (e.g., cataract, congenital retinal diseases, amblyopia, strabismus), anisometropia or astigmatism exceeding 1.5 D, a history of ocular surgeries, and systemic conditions (e.g., cardiac, endocrinological and respiratory diseases, or chromosomal abnormalities). **4.** Eligibility Criteria for MOSAIC (3 Years) Ireland, Australia 67 were eligibility criteria for MOSAIC Trial were age 6-16 years with spherical equivalent less than -0.50 diopters (D), no prior myopia therapy, no significant comorbidities, and no contraindications to atropine. **5.** Eligibility criteria for ATOM1 (2 Years) Singapore 55 were recruitment of participants was from children aged 6-12 years, refractive error of spherical equivalent between 1.00 D to 6.00 D in each eye as measured by cycloplegic autorefraction Anisometropia of spherical equivalent less than or equal to 1.50 D as measured by cycloplegic autorefraction Astigmatism of < 1.50 D or better in both eyes with normal intraocular pressure of 21 mmHg with no history of cardiac or significant respiratory diseases, no allergy to atropine, cyclopentolate, proparacaine, and benzalkonium chloride, no previous or current use of contact lenses, bifocals, progressive addition lenses, or other forms of treatment including atropine, no amblyopia or manifest strabismus, including intermittent tropia willing and able to tolerate monocular cycloplegia and mydriasis.

## 4. Safety and Tolerability Profile

### 4.1 Ocular Adverse Effects

The safety profile of low-concentration atropine is generally favorable, particularly when compared to higher (0.5-1.0%) concentrations. However, several important considerations warrant attention:

Visual Symptoms: Pupillary dilation (typically 1-2 mm with 0.01%, increasing to 2-3 mm with 0.05%) can cause photophobia (light sensitivity) and impaired near vision due to reduced accommodation amplitude. In the MOSAIC trial, 15% of 0.05% atropine users reported near vision blur, and 8% reported photophobia, whereas these symptoms were uncommon with the 0.01% dose (3% and 0%, respectively) 67. These effects are generally well-tolerated in children, particularly with photochromic lenses are used for outdoor activities and reading glasses are provided as needed for near work.

Intraocular Pressure (IOP) Concerns: Theoretical concerns exist regarding atropine's potential to elevate IOP, particularly through mechanisms involving trabecular meshwork obstruction by iris pigment released during pupillary movement or reduced trabecular tension secondary to ciliary muscle relaxation. However, a comprehensive 2024 review concluded that most studies show no clinically significant IOP changes in healthy children using low-concentration atropine 80. Nevertheless, caution is warranted in children with ocular hypertension or strong family history of glaucoma, as isolated cases of IOP elevation have been reported, including a documented case of a 7-year-old Chinese child who developed elevated IOP after unsupervised use of low-concentration atropine 81.

Ocular Surface Effects: Allergic reactions (e.g., conjunctival hyperemia, itching, and lid swelling) occur in approximately 3-5% of users, more commonly with higher concentrations and preservatives like benzalkonium chloride. Preservative-free formulations may reduce this risk. In the 0.03% atropine study, 35% of participants reported adverse events (mostly mild allergic reactions), with 16% discontinuing treatment due to tolerability issues despite no serious adverse events 68.

### 4.2 Systemic Safety and Long-Term Considerations

The systemic absorption of low-concentration atropine is minimal, with serum concentrations typically below detection limits. Consequently, systemic anticholinergic effects (e.g., facial flushing, dry mouth, urinary retention, constipation, and tachycardia) are exceptionally rare at concentrations ≤ 0.05%. Long-term follow-up data extending 10-20 years from the ATOM cohort provide robust reassurance regarding long-term safety: no increased incidence of cataracts, glaucoma, retinal detachment, or other visually significant complications were observed in former atropine users compared to matched controls 64. Similarly, European and Taiwanese studies that followed children using 0.01% atropine long-term found no serious adverse events attributable to treatment 82, 83.

### 4.3 Safety Management Recommendations

To manage the common side effects of atropine eye drops for myopia control, practical strategies focus on timing the dosage and using optical aids like photochromic or bifocal lenses (reviewed in 84, 85), including but not limiting: **1.** Managing Photophobia (Light Sensitivity): Photophobia is a common side effect caused by pupil dilation, usually mild, especially in the case of low concentrations of atropine (for example, 0.01%). **2.** Wear Sunglasses: They provide a simple way to reduce light sensitivity outdoors. **3.** Use of Photochromic Lenses Ware: Spectacle lenses darken in bright daylight, helping protection of children in indoor and outdoor activities. **4.** Tinted Glasses: For those sensitive to light, tinted glasses might be an option.

To manage near vision blur, use low-dose atropine to reduce amplitude of accommodation (reviewed in 84, 85). **1.** Alter Administration Time: Instilling the eye drops at bedtime is common for myopia control, which minimizes daytime blur as well as light sensitivity. **2.** Near Addition Glasses: When near blur is an issue, prescribe glasses with a reading addition, such as bifocal or progressive lenses, to support near work. **3.** Recommendation of Adequate Reading Distance: Encorage children to maintain an adequate reading distance and good lighting for near work. **4.** Combine with Optical Treatments: Atropine can be used in combination with multifocal contact lenses or orthokeratology, which can address both distance and near vision needs.

For administration of atropine (reviewed in 85), the methods include: **1.** Proper Instillation: Use punctal occlusion technique (applying light pressure to inside corner of an eye for 2 minutes after instillation) to reduce systemic absorption as well as potential side effects. **2.** Hand Hygiene: Wash hands before and after applying eye drops to prevent contamination. **3.** Monitor and Adjust Follow-up Visits: For example, 3-6 months intervals are necessary to monitor efficacy, assess side effects, and adjust the atropine concentration or other management methods as needed. **4.** Parental Help: Make sure that children are monitored when atropine drops are administered to ensure correct atropine eye drop application as well as safe storage.

## 5. Clinical Management Considerations

### 5.1 Patient Selection and Initiation Criteria

Appropriate patient selection is crucial for optimizing outcomes with atropine therapy. Ideal candidates typically include children aged 4-12 years with progressive myopia, defined as ≥ -0.50 D increase per year 86, particularly those with younger age of onset, family history of high myopia, or minimal time spent outdoors 87. The 2024 Expert Consensus on low-concentration atropine recommends initiating treatment when progression exceeds 0.50 D/year, though some specialists advocate earlier intervention in rapidly progressing cases or children approaching high myopia thresholds 84. Importantly, the landmark LAMP2 Trial demonstrated that 0.05% atropine significantly reduced myopia incidence (28.4% vs. 53.0% in placebo) when used preventatively in pre-myopic children with cycloplegic spherical equivalent between 0 and +1.00 D, suggesting potential for expanding indications to high-risk pre-myopes 22.

### 5.2 Practical Management Protocols

• Dosing Regimens: Once-daily evening instillation (one drop per eye) is standard, maximizing ocular contact time while minimizing light-related side effects. The MOSAIC Trial demonstrated excellent adherence (81%) with this regimen 67.

• Concentration Selection: Risk-stratified approaches are emerging: 0.05% may be preferred for rapid progressors (>1 D/year) or older children with less time for intervention 88, 89; 0.03% offers intermediate efficacy without significant effects 68; 0.01% provides maintenance therapy for young children without significant progression 90-92, such a concept is not all agreed 93. Ethnic considerations may apply, with some evidence suggesting higher concentrations are more effective in Asian populations.

• Monitoring Protocol: Comprehensive baseline assessment should include cycloplegic refraction, axial length measurement, visual acuity, accommodative amplitude, pupil size, IOP measurement, and anterior and posterior segment examination. Follow-up evaluations every 3 months should assess efficacy (refraction, axial length), side effects (accommodative amplitude, pupil size), IOP, and adherence 94. Optical coherence tomography (OCT) monitoring of retinal nerve fiber layer and macular thickness may be considered, though studies show no consistent atropine-related changes 95.

• Duration and Discontinuation: Treatment should ideally continue until mid-late adolescence (14-17 years), when myopia progression typically stabilizes. The ATOM follow-up demonstrated that early discontinuation (before age 14) is associated with greater rebound progression 57. Gradual tapering strategies (e.g., reducing concentration or frequency) over 6-12 months may mitigate rebound effects, particularly after higher concentrations 74.

The management of myopia is summarized in Table 4:

### 5.3 Combination and Alternative Therapies

• Synergistic Approaches: Atropine demonstrates additive or synergistic effects when combined with optical interventions like orthokeratology (OK) or defocus-incorporated multiple segments (DIMS) spectacles 96-99. Proposed protocols suggest instilling 0.01% atropine 5-15 minutes before OK lens insertion 100. Combination therapy may be particularly beneficial for rapid progressors insufficiently controlled by monotherapy.

• Alternative Agents: For children intolerant to atropine, pirenzepine gel (a relatively selective M1/M4 antagonist) demonstrated efficacy in early trials with reduced cycloplegic effects 101. 7-methylxanthine (7-MX), an adenosine receptor antagonist that increases scleral collagen synthesis, shows promise in oral formulation but remains investigational 102.

## 6 Unresolved Issues and Future Directions

### 6.1 Critical Knowledge Gaps

Despite substantial advances, several fundamental questions remain unresolved:

• Mechanistic Uncertainty: The precise cellular and molecular pathways through which atropine influences ocular growth require further elucidation. Particularly puzzling is the concentration paradox: how do minute concentrations insufficient for complete muscarinic blockade exert profound anti-myopic effects? Advanced techniques like single-cell RNA sequencing in primate models and proteomic analyses of human scleral fibroblasts may provide insights 40.

• Variable Treatment Response: Approximately 10-15% of children show minimal response ("non-responders") to atropine therapy 103. Predictive biomarkers—potentially genetic variants in muscarinic receptors, dopamine pathways, or scleral remodeling enzymes—could enable personalized therapy. Pharmacogenomic studies embedded in large trials are underway.

• Rebound Phenomenon: The post-cessation acceleration of myopia progression remains management challenge, particularly with higher concentrations and abrupt discontinuation. The biological basis of rebound—whether compensatory growth signaling or unmasking of suppressed progression—requires clarification to develop mitigation strategies 74.

• Prevention vs. Control: The promising LAMP2 results for 0.05% atropine in pre-myopes raise important questions: can atropine truly prevent myopia or merely delay onset? Long-term follow-up beyond adolescence is needed to determine whether delayed onset translates to reduced high myopia prevalence. It also remains unclear for the mechanism of action of atropine for pre-myopia prevention. Although successful interventions are true, the precise mechanism of some treatments, such as low-dose atropine, remains unclear. There is no definite consensus on the best approach to prevent pre-myopia. When is the best to initiate, combine, and discontinue treatments remains unknown. In addition, the long-term effectiveness and safety of atropine for pre-myopia prevention requires further investigation. Large scale and long-term clinical trials are needed for pre-myopia research. Studies, such as ATOM3 Trial for low-dose atropine, are critical for the efficacy and safety of such interventions for pre-myopic children. A precise understanding of atropine's mechanisms of action in pre-myopia is needed to guide development of novel mechanism-based treatments. In addition, personalized pre-myopia prevention strategies, based on combined factors including age, genetics, environment, and control methods, are needed. New research-based guidelines and strategies for effective interventions are required to maximize efficacy of atropine on pre-myopia prevention. Furthermore, known risk factors of pre-myopia such as ethnicity, age, genetics, and outdoor time require further investigation.

Rank of atropine clinical readiness is listed as below:

### 6.2 Emerging Innovations

Several promising developments may shape future clinical practice:

• Sustained-Release Delivery: Biodegradable implants, drug-eluting contact lenses, and punctal plug delivery systems could enhance adherence and provide steady drug levels, potentially improving efficacy and reducing peak-dose side effects. Early-phase clinical trials are evaluating a bimatoprost ring for sustained atropine delivery.

• Receptor-Specific Analogs: Compounds selectively targeting M1, M4, or dopamine D2 receptors may retain atropine's anti-myopic efficacy while minimizing unwanted effects like cycloplegia and mydriasis. Animal studies show promising results for compounds like pirenzepine and himbacine derivatives 104.

• Combination Formulations: Fixed-ratio combinations with dopamine agonists (e.g., apomorphine) or GABA enhancers might target multiple growth-signaling pathways simultaneously, potentially yielding synergistic effects that permit lower individual drug concentrations. Such approaches are in preclinical development.

• Personalized Concentration Titration: Adaptive dosing strategies using artificial intelligence algorithms that integrate individual progression rates, axial elongation velocity, and side effect profiles could optimize the benefit-risk ratio. Pilot implementation studies are exploring this precision medicine approach.

## 7. Conclusion

Low-concentration atropine has transformed the therapeutic landscape for childhood myopia, providing a safe, effective, and clinically practical intervention that slows axial elongation and reduces the risk of sight-threatening complications. The optimal concentration balances efficacy, tolerability, and rebound potential: 0.05% demonstrates superior efficacy in most trials, while 0.01% offers excellent tolerability with minimal rebound. Concentrations of 0.025-0.03% present promising intermediate options. Individualized management, taking into account age, progression rate, baseline refraction, and risk factors, remains essential. Ongoing research into mechanisms, alternative delivery systems, and combination therapies promises to further enhance outcomes. Ultimately, integrating atropine therapy with environmental modifications (e.g., increased outdoor time) and optical interventions offers the most comprehensive approach to combating the global myopia epidemic. As long-term safety data accumulate and novel treatment formulations emerge, atropine-based strategies will undoubtedly remain central to myopia management for the foreseeable future.

## Figures and Tables

**Figure 1:**
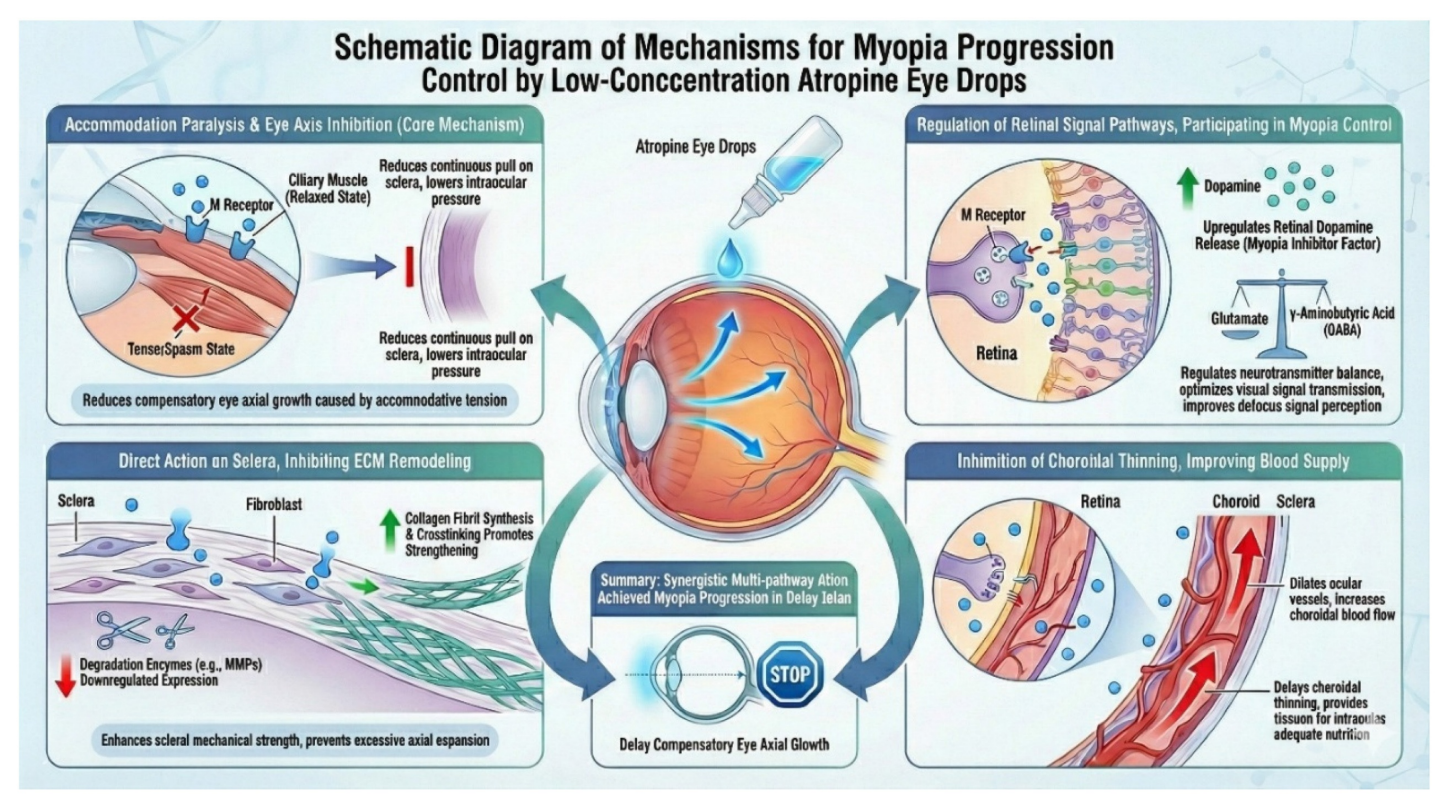
Summary of Current Myopia Control Mechanism.

**Table 1:** Summary of Key Mechanisms Implicated in Atropine's Anti-Myopic Effects

Biological Target	Proposed Mechanism	Experimental Evidence
Muscarinic Receptors (M1, M3, M4)	Regulation of scleral fibroblast activity and extracellular matrix remodeling	Upregulation of receptor mRNA in sclera; increased collagen synthesis 49
Choroidal Vasculature	Increased blood flow and thickness; improved retinal oxygenation	Enhanced choroidal perfusion in clinical studies; association with reduced axial elongation 50
Retinal Dopamine Pathways	Modulation of dopamine release and receptor signaling	Restoration of dopamine circadian rhythms in form-deprived models; synergy with L-DOPA 51
GABA Receptors	Activation of inhibitory GABAergic pathways	GABA agonists mimic atropine effects; GABA antagonists block atropine in animal models 42
Inflammatory Cytokines	Downregulation of pro-growth cytokines	Reduced TNF-α, IL-1β, and other cytokines in retinal/RPE tissue after atropine 52

**Table 2:** Comparative Efficacy of Different Atropine Concentrations in Major Clinical Trials

Atropine Concentration (%)	Trial, Duration and Location		Key Characteristics
0.01%	LAMP (1 Year), Hong Kong 65	n=438, After 1 year, the mean SE change was -0.27±0.61 D, with an increase in AL of 0.20±0.25 mm. The accommodation amplitude was reduced by 1.98±2.82 D. The pupil sizes under photopic and mesopic conditions were increased by 0.23±0.46 mm. All p<0.001.	
0.01%-0.02%	U.S. Trial (3 Years) 71	n=576, At month 36, 0.02% atropine slowed mean axial elongation (LSM difference, -0.08 mm; 95% CI, -0.13 mm to -0.02 mm; p = 0.005) while 0.01% atropine increased the responder proportion (OR, 4.54; 95% CI, 1.15-17.97; p = 0.03), slowed mean SER progression (LSM difference, 0.24 D; 95% CI, 0.11 D-0.37 D; p < 0.001), and slowed axial elongation (LSM difference, -0.13 mm; 95% CI, -0.19 mm to -0.07 mm; P < 0.001). Intermediate efficacy, Consistent effect over longer duration	
0.025%	LAMP (1 Year) Hong Kong 65	n=438, After 1 year, the mean SE change was -0.46±0.45 D, with a respective mean increase in AL of 0.29±0.20 mm. The accommodation amplitude was reduced by 1.61±2.61 D. The pupil sizes under photopic and mesopic conditions were increased respectively by 0.76±0.90 mm and 0.43±0.61 mm. All p<0.001.	
0.03%	Simonaviciute et al. (1 Year) Lithuania 68	n=72, After 1 year, a mean change by atropine in SE was -0.34 (0.44) D/year, lower than the -0.60 (0.50) D/year in the control group (*p* = 0.024). The change in AL was 0.19 (0.17) mm, compared to 0.31 (0.20) mm in the control group (*p* = 0.015). The atropine group had a significantly greater increase in ACD (*p* = 0.015). In total, 64.5% of patients in the atropine group showed progression <0.5 D/year, in contrast to 39.0% in the control group (*p* = 0.032).	
0.01%, 0.05%	MOSAIC (3 Years) Ireland, Australia 67	n=199, Compared with the group taking placebo then 0.05% atropine, the combined atropine then placebo groups had more spherical equivalent progression (adjusted difference, -0.13 diopters (D); 95% CI, -0.22 to -0.04 D; p = 0.01) and axial elongation (adjusted difference, 0.06 mm; 95% CI, 0.02-0.09 mm; p = 0.008), and the group taking 0.01% atropine then tapering 0.01% atropine had more axial elongation (adjusted difference, 0.04 mm; 95% CI, 0.009-0.07 mm; p = 0.04).	
1.0%	ATOM1 (2 Years) Singapore 55	n=346, After 2 years, the mean progression of myopia and of axial elongation in the placebo group was -1.20+/-0.69 D and 0.38+/-0.38 mm, respectively. In the atropine group, myopia progression was -0.28+/-0.92 D, whereas the axial length remained unchanged compared with baseline (-0.02+/-0.35 mm). The differences in myopia progression and axial elongation between the 2 groups were -0.92 D (95% confidence interval, -1.10 to -0.77 D; p<0.001) and 0.40 mm (95% confidence interval, 0.35-0.45 mm; p<0.001).	

*Compared to switch from 0.01% to placebo; SE = Spherical Equivalent; AL = Axial Length

**Table 3:** Safety and Tolerability Profile of Low-Concentration Atropine

Adverse Effect	0.01% Atropine	0.05% Atropine	Management Strategies
Photophobia (Light Sensitivity)	Uncommon (<5%)	Moderate (8-15%)	Photochromic lenses; sunglasses
Near Vision Blur / Accommodative Insufficiency	Mild (5-10%)	Moderate (15-25%)	Reading glasses for close work; monitor accommodative amplitude
Pupillary Dilation	Minimal (0.5-1.5 mm)	Moderate (2-3 mm)	Usually well-tolerated; cosmetic concerns rare
Allergic Conjunctivitis	3-5%	5-10%	Preservative-free formulations; switch to alternative concentration
Intraocular Pressure Elevation	Rare; no consistent pattern	Rare; isolated case reports	Baseline and periodic IOP checks; avoid glaucoma suspects
Systemic Effects	Extremely rare	Extremely rare	Nasolacrimal occlusion after instillation; wipe excess

**Table 4:** Myopia Management Protocols

Step	Myopia Management Stage		Key Actions and Protocols
1	Patient Identification and Risk Assessment		Perform comprehensive eye exams, such as cycloplegic refraction, keratometry as well as axial length measurement to determine true axial myopia.
			Identify and record various risk factors such as age onset, parental history, ethnicity, and outdoor activities.
			Educate parents and patients of risks of progressive myopia and goals of myopia management to slow down axial elongation.
2	Lifestyle and Environmental Adaptation		Encourage daily outdoor activities.
			Educate children of guidelines on near work, looking at distant objects from time to time, and increasing outdoors activities daily.
			Educate pupils to use appropriate reading distances (>33 cm) as well as adequate lighting.
3	Treatment Plan		Choose a practical treatment strategy based on progression rate, children age, practical lifestyle, and binocular vision status.
			Treatment: daily low-concentration atropine eye drops, spectacle lenses, multifocal soft contact lenses, orthokeratology, and their combinations.
4	Monitor and Follow-up		Schedule follow-up visits (for example, every 6 months for progressing myopes) to monitor efficacy and compliance.
			Monitor treatment efficacy by axial length measurements as well as cycloplegic refraction.
			Adjust the treatment when progression is off-target or when pronounced side effects occur.
5	Cessation and Tapering		Continue atropine treatment until myopia stabilizes, depending on individual variability in myopia stabilization.
			Tapering should be considered, and stabilization varies based on individual.
			

**Table 5:** Ranking Emerging Atropine Innovations by Clinical Readiness

Rank	Innovation	Clinical Readiness & Evidence Level	Feasibility Discussion
1	Sustained-release systems	High Readiness. These systems are in advanced clinical development, including Phase 3 trials and ongoing research. They have strong foundational evidence in overcoming limitations of traditional drops, such as low bioavailability and poor compliance.	Highly feasible and a transformative frontier in myopia management. They leverage existing contact lens technology to provide consistent drug delivery, potentially improving efficacy and adherence.
2	AI-powered dosing	Moderate Readiness. While AI is not yet used for *dosing*, the concept of individualized treatment based on patient response (e.g., age, ethnicity, progression rate) is widely discussed and implemented in clinical practice. Clinical data drives these personalized decisions (e.g., adjusting concentration from 0.01% to 0.05%).	Feasible as it relies on existing clinical data and algorithms rather than a novel drug or device. The challenge lies in standardizing protocols and ensuring consistent data input for AI optimization.
3	Receptor-specific analogs	Early-Stage Research. These are primarily in animal models and *in vitro* studies, aiming to target specific muscarinic receptors (M1/M4) involved in eye growth without affecting accommodation (near vision) or pupil size. This research is still foundational.	Less clinically ready, as the specific compounds need extensive preclinical testing before entering human clinical trials. The primary challenge is identifying a compound with the desired specificity and safety profile.
